# Supplementary material for: Are carbon emissions trading and green financial instruments synergistic? -Comprehensive quantitative research based on content analysis
Source: PLoS One. 2024 Mar 7;19(3):e0298601. doi: 10.1371/journal.pone.0298601 (PMC10919601; doi:10.1371/journal.pone.0298601)
Supplement: S1 Data — (ZIP) [file pone.0298601.s001.zip › Data/地级市Z_F工作报告.zip!/║■─╧/╗│╗»╩╨/└┤╫╘╣1⁄2╓┌║┼í╛┬φ┐╦╩2╛▌═°í┐_2017.docx]

各位代表：

现在，我代表市人民政府向大会报告工作，请予审查，并请各位政协委员和其他列席人员提出意见。

一、2016年奋起前行，各项工作稳中有进

过去的一年，我们坚决贯彻落实党中央、国务院和省委、省政府的决策部署，在市委的坚强领导下，紧紧围绕“一极两带”和“一个中心、四个怀化”战略，抢抓发展机遇，调动各方力量，奋力攻坚克难，推动经济持续回升向好，较好地完成了年初确定的各项目标任务。全年实现生产总值1390亿元（预计数，下同），增长8.1%；完成全社会固定资产投资1149亿元，增长14%；完成一般公共预算收入123亿元，增长11%；实现规模工业增加值305亿元，增长6.5%；实现社会消费品零售总额562亿元，增长11.8%；城乡居民人均可支配收入分别达到22560元、8000元，增长9%和11%。

对照市四届人大四次会议审查通过的《政府工作报告》，我们突出抓了七个方面工作。

1．突出抓了产业及产业园区发展。工业经济活力增强，完成园区基础建设投入58亿元，新建标准化厂房135万平方米，产业园区技工贸总收入、固定资产投资、招商引资到位资金均增长25%以上，税收增长22%以上；怀化工业园转型为省级高新技术产业园区，创建国家高新区通过专家现场考察；高新技术企业达47家、省级工程技术（研究）中心达10家；金益环保、合源水务等4家企业在新三板上市；出台新的《帮扶工业企业十条措施》，落实电价优惠和电力直供等政策，派驻25名特派员驻企帮扶。农业现代化步伐加快，完成粮食播种面积495万亩，新建标准化核心示范园区31个，新增省级现代化农业特色产业园区17个，新增省级重点龙头企业14家，中国驰名商标增至7个，农产品加工销售收入增长17%，推广种养殖业新品种新技术20项，农机总动力增长6%，沅陵县入列国家农业产业融合试点示范工程。第三产业发展迅速，入列全国物流标准化试点城市，一批重点商贸物流项目抓紧推进；阿里巴巴、京东等战略性电商企业落户怀化，苏宁易购中华特色·怀化馆、溆浦县农村商贸综合服务体电商试点上线运行，电子商务交易额达到104亿元、增长131%，怀化经开区获评全省电子商务示范基地。深入实施生态文化旅游“1354”工程，入列全国全域旅游创建示范区，签订张吉怀旅游共同体战略合作协议，全年建成国家4A级景区2个、3A级景区8个、国家四星级旅游饭店1家，接待游客4000万人次，实现旅游收入270亿元。怀化广告创意产业园在全省率先开园。成功举办武陵山片区健康产业博览会、湖南西部第八届汽车展销节和怀化市第十届房交会等展会活动。由于受工业品市场结构性萎缩、原材料价格上涨、传统产业转型升级困难等因素影响，规模工业增加值未能达到年初目标。

2.突出抓了城乡基础设施建设。鹤中洪芷生态城镇群建设步伐加快，高铁新城核心区完成规划设计，怀芷快速干线开工建设。中心城区基础建设扎实推进，高堰东路、云集西路、刘塘路、红星北路、顺天南路延长线、环城东路南延段、湖天大桥、锦溪桥竣工通车，高堰西路、北环线等项目进展顺利。完成城区道路补绿植绿20万平方米，城区重点楼盘、重点地段、重点单位、重点楼体亮化工程加快推进。城市管理全面升级，开展城区交通秩序、禁燃限燃、城区大气污染防治、户外广告、清街、渣土及建筑垃圾、查违控违拆违等专项整治。智慧城市云计算中心投入试运行。省级卫生城市复核居全省第2位，中心城区及洪江市、洪江区、会同、中方、芷江、新晃等县城通过省级文明城市创建验收。“美丽乡村·幸福家园”建设加快推进，建设“秀美村庄”200个。洪江安江镇成功创建省美丽示范乡镇，会同高椅村、芷江拾担村成功创建国家美丽宜居示范村庄，通道皇都侗文化村获评中国美丽休闲乡村。城乡交通网络不断完善，怀化铁路编组站搬迁、怀邵衡快铁、武靖高速、怀芷高速、怀化综合客运枢纽站、城市公交站场等项目加快推进，张吉怀高铁如期开工，怀化动车运用所正式获批。由于投资政策调整、前期工作难度超出预期，靖黎高速尚未开工，G319沅陵过镇改线工程仍在建设。

3.突出抓了脱贫攻坚。深入实施“四跟四走”精准扶贫，完成农村公路提质改造1094公里，解决农村33.8万人饮水安全问题，改造农村危房2.3万户，完成易地扶贫集中搬迁项目82个，辰溪照顶界村易地扶贫搬迁成为全省示范工程。落实农村贫困人口新农合参合费用全免政策。实施14个产业扶贫重点项目，完成257个贫困村特色产业培育，溆浦雪峰山旅游扶贫项目入列全国示范项目。全省教育精准扶贫试点市建设加快推进，11.07万名贫困学生获资助资金7657万元。完成农民工就业培训1.28万人次。建设金融扶贫服务站529个，发放扶贫小额信贷资金10亿元。洪江区提前脱贫摘帽，177个贫困村整体退出，18.81万贫困人口稳定脱贫，贫困发生率降至8.3%。

4.突出抓了生态保护和环境治理。大力实施“青山工程”，新增天然林保护工程500万亩，完成高速高铁沿线1002公里绿化和环城森林生态圈1650亩新造、4100亩补植补造、18.3万亩封山育林任务，新增沅陵、靖州国家森林公园2个。大力实施“碧水工程”，太平溪、氵舞水河综合治理工程加快推进，划定362处1000人以上集镇集中式饮用水源保护区，新增通道玉带河国家湿地公园1个。大力实施“蓝天工程”，全年空气质量优良率达88.2%，全省综合排名第2。大力实施“绿地工程”，新建市级综合性公园1个、街头小游园5个。实施99个重点减排项目，万元规模工业增加值能耗下降12%。

5.突出抓了重点领域改革创新。9大类34项重点改革稳步推进，简政放权、“三级一体”警务改革、“互联网+监督”、“互联网+民生服务”等改革经验在全省或全国推广。供给侧结构性改革有序推进，“三去一降一补”初见成效，关闭41家烟花爆竹企业、22家非煤矿山和4家煤矿企业，市本级消化商品房库存面积178万平方米。行政审批制度改革不断深化，成为全省精简幅度最大、保留事项最少的地区之一。市县两级公务用车改革同步实施。乡镇区划改革圆满完成。园区体制改革加快推进，人事管理、托管、容错免责等一系列政策措施相继出台。国企国资改革积极推进，经营性国有资产监管基本实现全覆盖。市本级出台《政府投资项目预算中介机构评审管理暂行办法》，管、评、审分离的监管机制进一步健全。麻阳全国农村信用体系建设试点和沅陵新型农村合作金融组织试点有序推进。农村产权抵押贷款和融资担保模式不断创新，两权直接抵押贷款等模式有效推行。启动城市公立医院综合改革，取消药品加成。出台《加快科技服务业发展八条措施》，文化创意孵化园、大学生创业园创客社区、科技型孵化器达到5家，新增国家级创新平台2个。长沙海关驻怀化监管机构正式开办业务。深化商事制度改革，新登记市场主体3.65万个，增长36%。国家工商总局商标注册业务怀化受理中心正式启用。成功引进传化物流公路港等一批重大项目，招商引资到位资金426亿元。

6.突出抓了民生实事。民生保障力度不断加大，民生支出占财政支出比例达78.5%。省市确定的25项重点民生实事全面完成。新增城镇就业3.58万人、农村劳动力转移就业4.7万人，城镇零就业家庭实现动态清零。农民工保证金制度全面推行，社会保障覆盖面进一步扩大，五大保险参保登记达560万人次。在全国率先将“三无六零”等特殊困难群体全部纳入政策保障范围，建设留守老人和留守儿童关爱服务中心500个，改扩建乡镇敬老院6所。残疾人两项补贴全面启动，发放资金5268万元。40个基层儿童福利服务体系试点建设扎实推进。开工棚户区改造3.48万套、公租房5063套。

7.突出抓了各项社会事业。建设义务教育合格学校160所、农村公办幼儿园38所，岳麓欧城小学、宏宇初中、长郡怀化分校、芷江师范一期工程进展顺利。全面完成卫生惠民工程，医疗服务水平明显提升。认真实施全面二孩政策，计划生育工作稳步发展。完成村级综合文化服务中心建设100个、广播电视“户户通”工程11.88万户。深入开展“欢乐潇湘·幸福怀化”等30余次大型群众文化活动。完成第三次农业普查综合试点工作。成功组织纪念红军长征胜利80周年、新晃60周年县庆等重大活动。精心承办湖南省第七届全民健身节。两名运动员在里约残奥会荣获4金1银优异成绩，实现怀化奥运史上金牌零的突破。开展信访积案集中化解，包案化解重点信访积案71件；受理网上信访事项3453件，按期办结率100%。扎实推进社会治安防控体系建设三年计划，公众安全感得到提升。全面开展“大排查大管控大整治”专项行动，安全生产形势总体平稳可控。扎实开展食品药品领域专项治理，食品药品安全保障有力。推进法治政府建设，依法行政水平不断提升。办理人大代表建议188件、政协委员提案163件。推进国防教育和国防动员工作，预备役部队和民兵建设水平不断提升，第七次荣获“全国双拥模范城”称号。工会、共青团、妇女儿童、科学普及等各项事业健康发展，审计、监察、档案、质监、水文、气象、地方志、水库移民、防震减灾、民族宗教、老龄老干、优抚安置、住房公积金等工作取得新成效。

二、四年共同奋斗，怀化站上了发展新起点侯卫东官场笔记

2016年主要目标任务的实现，标志着市第四届政府各项工作顺利收官。回顾四年来的工作，我们克服多重困难和挑战，干成了一批大事，办好了一批实事，攻克了一批难事，怀化的发展翻开了新的一页，开启了追赶超发展的新征程。

这四年，发展战略实现大提升。精准把握和全面落实“五位一体”总体布局、“四个全面”战略布局、“五大新发展理念”的本质要求，积极抢抓“一带一路”、“一核三极四带多点”等重大机遇，深入实施“一极两带”和“一个中心、四个怀化”战略，使怀化由发展边缘地带上升为增长极地带，在全省发展格局中的地位实现历史性跨越、转变和提升，怀化未来的发展从此站上了全新的历史起点。

这四年，交通条件实现大改善。2014年底沪昆高铁怀化段建成通车，怀化进入“高铁时代”，高铁、高速公路通车里程居全省首位，芷江机场通航多个城市。便捷的现代立体交通网络，让怀化融入了全国发展的大格局，大西南综合交通枢纽地位更为稳固。中心城区建成区面积扩展到65平方公里、道路骨架拉开到90平方公里、常住人口达到62万，城镇化率由2012年的39.0%提高到45.4%。一座欣欣向荣、宜居宜业的生态中心城市正在加速崛起，怀化在五省边区的区位优势更加凸显。

这四年，产业建设实现大进步。把产业及产业园区建设作为第一大主战场，强力推进倍增计划，强化现场巡检讲评，形成了以产业强实力、凭产业论英雄的浓厚氛围，产业意识大为增强。完成园区基础设施建设投资165亿元，建成标准厂房300万平方米，创新园区管理体制和运行机制，推动优势特色产业向园区集聚，怀化高新区、怀化经开区和13个省级工业集中区活力不断增强，产业平台明显优化。科学把握怀化相对优势和资源禀赋，主抓现代商贸物流、生态文化旅游、医药健康、绿色食品四大特色支柱产业的思路越来越清晰，产业特色更加鲜明。规模工业增加值年均增长7.5%，三次产业结构由14.5：44.9：40.6调整为14.5：39.5：46.0，产业园区技工贸收入达到2947亿元，怀黔千亿工业经济走廊和一批千亿、百亿园区加快规划建设，产业实力稳步提升。

这四年，脱贫攻坚实现大突破。实施最严格的军令状管理考核，开展县（市、区）党政正职脱贫攻坚述职考评，脱贫攻坚责任进一步压实。积极探索“四跟四走”新思路，全面推进驻村帮扶、结对帮扶和社会帮扶，深入开展金融扶贫、产业扶贫、教育扶贫、健康扶贫、园区扶贫和生态移民扶贫，脱贫攻坚措施进一步精准。整合投入资金50亿元，实施“十个到村到户行动”和精准扶贫“十大工程”，落实扶贫项目1.1万个，覆盖农村困难人口270余万人次，脱贫攻坚项目进一步抓实。贫困人口由2013年的90.23万减少到35.62万，贫困发生率下降12.7个百分点，易地扶贫搬迁3.28万人，脱贫攻坚实效进一步显现。

这四年，干部作风实现大转变。通过深入开展党的群众路线教育实践活动、“三严三实”专题教育和“两学一做”学习教育，大力开展“宣战庸懒散、提振精气神”作风大整治，各级干部精神面貌焕然一新，干事创业积极性空前高涨。扎实推进文明乡村、文明社区创建，2014年、2015年社会治安综合治理工作和社会治安综合治理民意调查名列全省前茅，政治生态、社会风气明显好转。

四年来的成就，为今后发展打下了坚实基础；四年来的实践，为继续前进积累了宝贵经验。

必须坚持贯彻落实上级精神与推动怀化实际工作相结合。自觉将怀化的改革发展置于全国、全省发展大局中去谋划和推动，紧跟中央重大战略思想、方针政策和省委、省政府决策部署，紧密结合怀化特点和工作实际，创造性抓好贯彻落实，全力推动上级精神和工作部署在怀化落地生根、取得实效，并努力创造怀化的特色和经验。

必须坚持科学谋划与精准落实相结合。坚持运用战略眼光、科学理念、现代思维谋划怀化发展，对产业及产业园区发展、中心城区综合提质、决战决胜脱贫攻坚等事关全局的重大事项进行精心布局、周密部署，确保方向正确、导向明确。在此基础上，把推动落实作为政府工作的根本着力点，精准分解各项工作及指标责任，强化高频调度和警示约谈，以踏石留印、抓铁有痕的作风，确保工作落地见效。

必须坚持整体推进与重点突破相结合。紧紧抓住发展这个第一要务，从全市发展大局、全体人民利益出发思考问题、履行职责，统筹抓好政府各项工作，切实做到十个手指弹钢琴、全市上下一盘棋。与此同时，以敢啃硬骨头、敢于涉险滩的担当精神，抓住牵一发而动全身的投融资体制改革、城管重心下移、国家高新区申创等“牛鼻子”工程，通过核心领域、重点工作、关键环节的突破，带动整体工作迈上新台阶。

必须坚持自主奋斗与借力而为相结合。把激发内生动力作为推动怀化发展的决定性力量、根本性举措，积极应对各种矛盾困难，在发挥自身优势中找出路，从挖掘自身潜力上下苦功，扎实开展化危为机解难题集中攻坚系列行动，奋力扭转了经济下行势头。与此同时，注重借力发展、借梯登高，立足怀化处在中西部地区结合部、新丝绸之路和湖南“一带一部”重要节点上的区位特点，积极争取上级重视和政策支持，积极搭建开放合作和创新创业平台，汇聚更多力量、集聚更优资源推动怀化追赶超发展。

必须坚持发挥优势与补齐短板相结合。正确看待、倍加珍视怀化的生态、交通、区位等比较优势，持之以恒把优势放大、做足、用活，使之成为推动怀化发展的优质资源，切实做到优势优先发展。同时，牢固树立短板也是机遇的理念，千方百计补齐长期制约发展的产业、城建、扶贫、开放等短板，使之成为推动怀化发展的新增长点，切实做到短板拉长发展。

各位代表！回首这一路走来的四个春秋，我们在各类问题集中凸显、各种矛盾相互交织的重重困难中奋起前行，我们为扭转经济持续下行、主要指标排位靠后的态势而奋力拼搏，我们与突如其来的自然灾害、长期延续的深度贫困作奋勇抗争。四年的艰辛努力和顽强奋斗，为怀化的长远发展打下了坚实基础。在这里，我们要深深感谢省委、省政府和市委的科学谋划、坚强领导，深深感谢各级人大代表、政协委员和各民主党派、工商联、无党派人士的倾力支持、有效监督，深深感谢驻怀部队指战员、武警官兵和中央、省驻怀单位的辛勤工作、无私奉献，深深感谢525万怀化干部群众团结一心、共同拼搏。我们深深知道，因为有你们，怀化得以创造历史；我们更加坚信，只要有你们，怀化还将赢得未来！

在肯定成绩的同时，我们也清醒地看到发展中还存在不少问题，主要是：经济总量不大、实力不强，产业及产业园区短板明显，与怀化“一极两带”的新定位还不相适应；经济结构不优，自主创新能力不强，节能减排和环境保护任务较重，与我们发挥生态优势、建设生态中心城市的目标还不相适应；城乡发展不平衡，就业、社保压力大，脱贫摘帽时间紧迫、任务艰巨，与人民群众改善生活水平、共享发展成果的期待还不相适应；少数干部精神不振、执行力不强，不作为、慢作为甚至乱作为，发展环境不优问题依然存在，与怀化追赶超发展的要求还不相适应。我们将认真负责地正视这些问题，不回避、不推卸、不护短，下最大决心，尽最大努力加以解决。

三、决战决胜未来五年，坚决完成“三大历史使命”

今后五年，是我市脱贫攻坚、全面小康的决胜阶段，是基本奠定湖南新增长极地位的关键时期。当前，世界经济在深度调整中曲折复苏，新常态下我国经济发展长期向好的基本面没有改变，供给侧结构性改革红利正逐步释放。随着国家“一带一路”和湖南“创新引领、开放崛起”等系列重大战略、重大改革、重大政策、重大举措深入实施，怀化的战略地位将更加突出，政策优势将不断聚集。随着“一极两带”和“一个中心、四个怀化”建设的加快推进，全市干部群众心齐气顺、风正劲足的氛围日益浓厚，我市将进入大有可为的黄金发展期。站在新的历史起点，我们要以迎接撤地建市20周年为契机，准确把握发展机遇与自身优势，奋力推动怀化新一轮大开放、大建设、大发展。

未来五年总体目标就是坚决完成“三大历史使命”：提前实现全面脱贫摘帽，到2019年13个县（市、区）全部脱贫摘帽，贫困村全部退出，建档立卡贫困人口全部脱贫；如期全面建成小康社会，全市全面小康实现程度达到95%以上；基本奠定湖南新增长极地位，主要经济指标增速保持全省前列，综合经济实力大幅提升。

实现上述目标，必须加快构建五大体系。

——围绕创新驱动，加快构建科技创新体系。依托怀化高新区等创新载体，进一步集聚各类要素资源，努力建成五省边区绿色发展创新驱动示范区。加强科技创新平台建设，建成一批研发中心、孵化基地、实验室。强化企业创新主体地位，培育更多具有竞争力的创新型领军企业。积极构建政产学研用有机结合、科工贸一体化的创新体系，加快科研成果转化和产业化，有效发明专利达到1000件以上。建立财政10%稳步增长投入机制，发挥科技专项资金的杠杆作用，撬动社会资本、金融资本向科技创新聚集。深化科技体制改革，强化科技创新体系、科技服务能力建设，深度推进创新链、产业链、资金链、价值链“四链融合”。加快培养引进高精尖创新创业人才，大力推进大众创业、万众创新，让创业创新在全社会蔚然成风。

——围绕转型发展，加快构建现代产业体系。扎实推进供给侧结构性改革，抓实“三去一降一补”五大任务。全力抓好怀化国家高新区创建，到2019年技工贸收入突破1600亿元，以此为龙头带动怀黔千亿工业走廊、13个省级工业集中区加快发展，建设一批百亿产业、百亿园区、百亿基地。加快推进武陵山现代电子商务产业园、武陵山商贸物流城、中央商务区、高铁南站现代商业街区、怀化现代粮食物流中心、佳惠物流配送中心等重大项目建设，大力发展现代商贸物流业。深入实施“1354”工程，下力气发展全域旅游，积极保护和开发古城古镇古村资源，抓紧申建5A级景区，大力发展生态文化旅游业。突出发展中药材种植加工、健康养老养生服务业，加快怀仁博世康医药物流健康产业园、靖州茯苓科技产业园、新晃龙脑健康养生园、麻阳长寿产业园建设，大力发展医药健康产业。依托怀化国家农业科技示范园区、靖州国家现代农业示范区、新晃国家地理标志产品保护示范区，建设一批绿色食品基地，大力发展绿色食品产业。加快发展先进制造、文化创意等战略性新兴产业和电子信息、云计算等新一代信息技术产业。推进农业供给侧结构性改革，抓好土地流转、农田水利、农业科技等各项工作，全面落实省三个“百千万”工程，实施优质农产品品牌战略，扶优扶强农业产业化龙头企业，加快推进农业现代化。全面推进供销社综合改革，积极培育农业新型经营主体。大力发展电子商务、社区服务、住宅产业等现代服务业。加快发展现代金融业，努力把怀化建成五省边区金融中心。大力推进“质量强市”战略，推动怀化产品向怀化品牌转变。深入实施“互联网+”行动计划，推动互联网与实体经济深度融合。突出抓好招商引资，千方百计引进战略投资者。积极培育企业主体，大力支持中小微企业发展。

——围绕城乡统筹，加快构建新型城镇体系。坚持规划引领，以沪昆高铁经济带为东西轴，以张吉怀精品生态文化旅游经济带为南北轴，统筹推进鹤中洪芷核心引领区、北部生态保护与特色产业发展区、南部生态文化旅游先导区、西部湘黔经济合作带示范区建设，加快构建“四大功能区”发展新格局。实施以高铁南站为核心的高铁新城建设工程，到2021年高铁新城建成区面积达到15平方公里，中长期达到34平方公里。实施以公共设施配套为重点的中心城区综合提质改造工程，加快新城建设和旧城改造，推进城市应急备用水源、背街小巷、农贸市场改造、垃圾和污水处理、城市公交站场等配套设施建设，提升城市品质品位。实施以城市干线公路为重点的鹤中洪芷生态城镇群建设工程，加快怀芷快速干线、怀洪旅游干线建设和怀黔城际干线改造，启动城市轨道交通前期工作，打通鹤中洪芷融合发展通道。到2021年，城镇群建成区面积达150平方公里，人口达150万。实施以铁路建设为重点的基础建设工程，突出抓好一批重大交通、能源、水利等基础设施项目。实施以太平溪、氵舞水河流域综合治理为重点的生态建设工程，开展大规模国土绿化和城市增绿行动，高品质建好城市主题公园，努力建设中西部重要生态安全屏障。实施以易地扶贫搬迁为重点的县乡城镇建设工程，全面完成11.03万人易地扶贫搬迁和18万户农村C、D级危房改造任务。以争创省级园林城市、国家环保模范城市、国家森林城市为抓手，全面提升城市管理科学化、规范化、精细化、特色化水平。到2021年城镇化率提高到55%以上。

——围绕开放引领，加快构建综合交通枢纽体系。加快打通国际贸易大通道，开行怀化点对点直达中欧班列，努力把怀化建设成为中欧铁路通道上的重要节点；争取张吉怀高铁南延至桂林，开通怀化至东盟铁路联运线，依托北部湾实现借船出海；争取建设靖永郴铁路，依托沪昆高铁、沪昆铁路和沪昆高速公路等交通干道，打通南亚次大陆国际贸易通道。对接和融入省内两小时高铁经济圈和“七纵七横”高速公路网，着力提升对外通达水平。加快完善市域内路网建设，实现高铁、高速、国省干线、城市公交、农村公路的有效对接。积极推进大通关建设，完善提升公路港、铁路港、航空港等平台，加快建设物流保税中心。携手武陵山片区、贵州共同开发精品旅游线路，共建高铁经济带。

——围绕脱贫致富，加快构建民生保障体系。切实强化“悠悠万事、脱贫为大”的责任感和紧迫感，全面深入推进“四跟四走”精准扶贫，以超常规手段攻克脱贫难关。到2019年13个县（市、区）全部脱贫摘帽，848个贫困村全部退出，35.62万贫困人口全部脱贫；到2020年全面小康实现程度达到95%以上。落实更加积极的就业政策，突出抓好高校毕业生、退伍军人、城镇就业困难人员、农村富余劳动力的就业创业工作。推进五大社会保险扩面征缴，加强城乡低保提标、医疗救助、养老服务体系建设。积极发展社会福利、社会慈善、社会公益事业，加大社会救助力度，抓好“三无六零”人员脱贫兜底、五保供养、孤儿保障、灾民救助工作。健全符合实际的住房保障和供应制度。优化教育教学资源配置，加强教师队伍建设，促进各类教育均衡优质发展，使教育发展水平与城镇化进程相适应。推进芷江师范升专工作。深化以城市公立医院综合改革为重点的医药卫生体制改革，积极实施医疗卫生服务能力提升工程，加快建设五省边区医疗卫生服务中心。落实全面二孩政策，强化公共服务保障，促进人口均衡发展。推进市县文化体育设施建设，完成2769个村（社区）综合文化服务中心建设。加强民族文化挖掘保护，支持侗寨申遗。广泛开展全民健身运动，提高全民健康素质。加强社会综合治理和安全生产工作，不断增强人民群众的安全感。

四、全力奋战2017年，确保各项工作开好局起好步

今年是实施“十三五”规划的重要一年，是供给侧结构性改革的深化之年，也是新一届政府任期的第一年，必须全力以赴开好局、起好步。总体要求是：全面贯彻党的十八大和十八届三中、四中、五中、六中全会精神以及中央经济工作会议、省党代会、省委经济工作会议精神，统筹推进“五位一体”总体布局，协调推进“四个全面”战略布局，坚持稳中求进工作总基调，牢固树立和贯彻落实新发展理念，适应把握引领经济发展新常态，坚持以提高发展质量和效益为中心，坚持以推进供给侧结构性改革为主线，坚持创新引领、开放崛起，奋力推进“一极两带”和“一个中心、四个怀化”建设，全面做好稳增长、促改革、调结构、惠民生、防风险各项工作，打好脱贫攻坚、转型升级、环境治理战役，促进经济平稳健康发展和社会和谐稳定，以优异成绩迎接党的十九大胜利召开。主要目标任务是：地区生产总值增长8.5%左右，一般公共预算收入增长11%左右，规模工业增加值增长7%左右，全社会固定资产投资增长17%左右，社会消费品零售总额增长12%左右，城乡居民人均可支配收入分别增长9%、11%以上，单位GDP综合能耗及主要污染物减排完成省里考核目标。

（一）坚持以招商引资为抓手，持续推进产业及产业园区建设。强力招商引资。积极对接全省开放崛起发展战略，加强与长三角、珠三角、成渝城市群等地的深度对接，找准市场需求和我市优势的结合点，抓好项目、产业链、产业集群点线面结合，提高项目招商精准度、成熟度。坚持全市一盘棋，开发包装一批重大招商项目，统筹招商项目布局。点对点抓好央企、大企业集团和重点商会的对接，突出引老乡、回故乡、建家乡。引进内外资总额600亿元以上。优化园区平台。围绕建设五省边区绿色发展创新驱动示范区，加强衔接跟踪，力争上半年获批国家高新区。推进园区人事、财税和投融资体制改革，探索建立“园政合一”管理新模式。完成园区基础设施投资68亿元，实现产业园区技工贸总收入1245亿元。推进产业升级。积极对接“湖南智造”战略，实施重点产业项目126个，其中四大特色支柱产业项目59个。重点抓好智慧城市建设、农网升级改造、新能源汽车等项目，完成工业投资355亿元，增长15%。推进中方葡萄、靖州杨梅、麻阳冰糖橙等基地建设，发展茯苓、龙脑樟、雪峰天麻等道地中药材。大力开展无公害农产品、绿色食品、有机农产品和地理标志农产品认证，创建一批特色农产品品牌。新增市级以上农业产业化龙头企业15家，农产品加工销售收入增长16%以上。加快武陵山现代商贸物流城、华美立家武陵国际商贸城等重点项目建设，举办好车展节、车博会和武陵山片区生态食博会。大力发展电子商务，积极实施电子商务示范培育工程，电子商务交易额突破200亿元。全面实施国家全域旅游示范区创建，加快融入张吉怀精品生态文化旅游经济带，开发一批精品旅游线路，建设一批特色文化精品景区，积极创建5A级景区，旅游总收入增长20%以上。

（二）坚持以中心城区和县城建设为龙头，统筹推进城乡协调发展。加快高铁新城建设。推进怀化综合客运枢纽站、高铁新城地下综合管廊和路网、大湘西游客集散中心等项目建设，加快一批中高档住宅建设。加快中心城区综合提质。突出抓好新一轮太平溪和氵舞水河综合治理、迎丰路立交桥、城区公共停车场等重点项目。深入推进城区重点楼盘、重点地段、重点单位、重点楼体亮化工程。扎实抓好太平桥片区棚户区改造、人民西路暨鹤州路片区旧城改造和城中村棚户区改造，加大拆违控违力度。深化城管体制改革，依托智慧城市平台，推进交通管理、环卫保洁智能化，实现城市管理全面升级，成功创建省级园林城市。加快县城建设。坚持优势优先发展，积极建设北部生态特色产业县城、南部生态文化旅游县城和西部湘黔经济合作示范县城，推动建制镇彰显特色、竞相发展、优势互补。加快美丽乡村建设。深入推进“百城千镇万村”新农村建设工程，扎实抓好美丽乡村示范村建设，深化农村环境综合治理，让广大农村山更青、水更绿、天更蓝。

（三）坚持以易地搬迁和产业扶贫为重点，奋力打好脱贫攻坚战。把脱贫攻坚作为首要政治任务和第一民生工程，实现17.68万人脱贫、419个贫困村出列。扎实抓好易地扶贫搬迁。开展易地扶贫搬迁攻坚年行动，投资31.89亿元，安置5.8万人。落实搬迁群众创业就业、产业发展、技能培训等后扶政策，确保搬迁一户、脱贫一户。深入推进“四跟四走”精准扶贫。建立资金多元投入、政策多元整合机制，确保投入扶贫资金10亿元以上。实施扶贫产业项目3800个，建成村级扶贫产业园419个。完成农村公路提质改造1200公里、农村饮水巩固提升23.8万人、集中改造农村危房3万户以上。建立市县乡村组五级管理机制，层层压实帮扶责任。认真抓好后续跟踪服务，强化对脱贫对象的后续帮扶，确保不因病、因学、因灾返贫。

（四）坚持以供给侧结构性改革为引领，不断提高经济发展的竞争力和开放度。深入实施供给侧结构性改革。妥善处置“僵尸企业”，加快淘汰煤炭、电解锰等落后产能。抓紧房地产遗留问题处理，积极培育住房租赁市场，加大棚改货币化安置力度，多途并举推动房地产去库存，确保取得明显成效。强化政府性债务管控，依法整治非法集资，防范化解金融风险。千方百计降低企业成本及税费负担。大力推进创新驱动发展。把科技创新摆在发展全局的核心位置，大幅度增加财政投入，确保全社会科技研发经费突破14亿元。新增高新技术企业8家，产值和增加值增长20%。科技企业孵化器通过省级认定。加快科技成果转化，实施一批重大科技专项，重点支持医药健康、绿色食品等特色产业关键技术研发，建立市级以上科技创新平台4个。加速提升对外开放水平。推进怀邵衡快铁、渝怀铁路二线、张吉怀高铁、怀化铁路编组站迁建和动车运用所建设，启动焦柳铁路怀化至柳州段电气化改造，全面做好怀化至桂林高铁等项目前期工作，推进芷江机场航站楼改扩建，推动靖黎、沅辰、溆洞等高速公路项目开工。提升长沙海关驻怀化机构运行效率，发挥商标注册怀化受理中心辐射作用。发展对外经贸，培育外贸龙头企业，鼓励本土企业走出去，支持境外承揽工程，扩大劳务输出规模。

（五）坚持以增强群众获得感为目标，优先保障各项民生事业。继续稳定和扩大就业。实施积极的就业政策，加快推进公共就业服务平台标准化建设，新增城镇就业3.5万人，登记失业率控制在4.5％以内，新增农村劳动力转移就业3.8万人。积极完善社会保障体系。强力推进社保扩面征缴，扩大社会保险覆盖面。深入实施医保基金总额控制付费方式改革，加强城乡居民养老保险后续服务，积极探索完善失地少地农民社会保障制度。加快各项社会事业发展。坚持教育优先发展，调动各类资源，全面完成城区教育发展第二个五年行动计划。加快城区中小学校建设步伐，新增城区（含县城）义务教育学位1.9万个，稳步改善农村义务教育薄弱学校办学条件；基本完成芷江师范搬迁，基本建成职教基地“两馆一中心”。深化医药卫生体制改革，协调推动医疗、医保、医药联动改革；全面实施怀化“智慧医疗”建设，落实公立医院互联互通工作；抓好公共卫生、妇幼健康服务和疾病防控工作。加快中心城区湖天群众文化展演中心建设，推进县市区公共文体设施、村（社区）综合文化服务中心建设。广泛开展全民健身运动，大力发展体育产业，办好全国柔道锦标赛和第五届市运会。支持靖州、芷江办好30周年县庆活动。加强双拥共建、国防动员、民兵预备役、人民防空等工作，推动军民深度融合发展。统筹抓好民族宗教、外事侨务等工作，加强审计、监察工作，促进统计、残联、档案、保密、气象、水文、地方志、防震减灾等各项事业全面协调发展。

（六）坚持以创新社会治理方式为主线，有效促进社会和谐稳定。加强公共安全体系建设。落实社会稳定风险评估制度，完善社会治安防控体系建设，强化情报预警研判和重点人员管控，有效防范和化解各类风险。重视网络舆情监管，提高新媒体时代舆情引导水平。广泛开展平安创建活动，严密防范和依法打击各类违法犯罪。深入开展食品安全“四小”和市场失信等集中整治，确保群众吃上放心食品、用上良心药品。强化消防安全隐患整治，严格公共场所安全管理，突出抓好中心市场、农村团寨等重大火灾隐患专项整治，坚决遏止群死群伤重大安全事故发生。推动普法和依法治理。抓好“七五”普法，进一步推进全市依法治理工作，提升社区矫正安置帮教、人民调解工作水平，切实抓好人民调解组织和人民调解员队伍建设，落实县乡村三级矛盾纠纷排查调处制度。坚决守住安全生产底线。突出落实企业安全生产主体责任、政府及相关部门的安全监管责任，强化安全生产工作目标管理。继续保持打非治违高压态势，依法惩处各类非法违法行为。加强应急救援工作，提高应急处置能力，科学应对处置各类突发事故。

（七）坚持以奋发作为谋发展为导向，切实加强政府自身建设。严守政治规矩，确保中央政令畅通。政府各级干部特别是领导干部要牢固树立“四个意识”，坚决维护以习近平同志为核心的党中央权威，做到党中央制定的方针政策必须执行，党中央确定的改革方案必须落实，坚决按照省委省政府和市委的工作要求，推动政府各项工作规范、高效、有序运转。坚持依法作为，规范权力运行。自觉接受人大法律监督、工作监督和政协民主监督，坚决执行人大及其常委会的决议决定，认真听取各民主党派、工商联、无党派人士和人民团体意见。健全依法决策机制，实施信访评估、社会稳定风险评估、行政决策风险评估“三评合一”，落实公众参与、专家论证、集体讨论决定重大行政决策法定程序，实行重大决策终身责任追究制度。坚决履行党政主要负责人推动法治建设职责，着力建设法治政府。坚持主动作为，强化责任担当。始终把干事创业谋发展作为最大责任，敢于直面发展中的问题和矛盾，以不回避、不掩盖、不畏难的态度，积极主动作为，坚决纠正懒政、怠政和为官不为现象。强化政风政纪督察，对不守信、乱作为的政府部门及工作人员，严格执纪问责，最大限度优化政务环境、提高行政效率。各级政府及其工作人员要有为了怀化发展不计个人得失的胸怀，勇于负重、实干克难，不怕承担责任，不怕承担风险，推动工作雷厉风行，促进发展永不懈怠。要按照“三个区分开来”的要求，建立容错纠错机制，宽容干部在工作中特别是改革创新中的失误，旗帜鲜明地为敢于担当的干部担当，为敢于负责的干部负责。坚持持久作为，务求一抓到底。蓝图一旦绘就，路子一旦选准，就要放开手脚干、顶着压力上，咬定目标不放松，不达目的不罢休，以扎硬寨、打硬仗的精神一抓到底。要把抓执行作为政府工作的生命线，对确定的目标、部署的工作，务必起而行之、锲而不舍，做到事有专管之人、成有限定之期。健全工作激励机制和督查问责机制，层层量化指标、细化任务，持之以恒地推动重点工作落到实处。坚持廉洁作为，弘扬清风正气。把纪律和规矩挺在前面，严格执行廉洁自律各项规定，严守“两准则、四条例”，从严落实“两个责任”和“一岗双责”。强力正风肃纪，以严格问责倒逼机关作风持续转变，扩大暗访和作风评议范围，严查群众身边的腐败问题，努力实现干部清正、政府清廉、政治清明、社会清新。

各位代表，怀化的昨天精彩纷呈，怀化的今天生机盎然，怀化的明天希望无限。让我们更加紧密地团结在以习近平同志为核心的党中央周围，在省委省政府和市委的坚强领导下，紧紧依靠全市各族人民，把使命牢记在心，把责任扛上肩膀，把机遇变为现实，不忘初心、继续前进，为加快建设“一极两带”和“一个中心、四个怀化”作出新的更大贡献！
